# The biological dimensions of transcendent states: A randomized controlled trial

**DOI:** 10.3389/fpsyg.2022.928123

**Published:** 2022-09-08

**Authors:** Dawson Church, Amy Yang, Jeffrey Fannin, Katharina Blickheuser

**Affiliations:** ^1^National Institute for Integrative Healthcare, Petaluma, CA, United States; ^2^Northwestern University Feinberg School of Medicine, Chicago, IL, United States; ^3^Thought Genius LLC, Peoria, AZ, United States

**Keywords:** brain waves, cortisol, immunity, anxiety, depression, group therapy, meditation, transcendent states

## Abstract

This study evaluated the biological dimension of meditation and self-transcendent states. A convenience sample of 513 participants was drawn from attendees at a 4-day guided meditation workshop. Half were randomly assigned to an active placebo control intervention. All were assessed on a variety of measures, both psychological [anxiety, pain, posttraumatic stress disorder (PTSD), positive emotions, and transcendent states], and physiological (physical functioning). Additional biological assessments including salivary immunoglobulin-A (SIgA), cortisol, and Quantitative Electroencephalography (qEEG) were obtained from subset of the Experimental group (*N* = 117). No significant difference in psychological symptoms or positive emotions was observed between Experimental and placebo groups at baseline. At post-test, significant improvements were noted in the Experimental group, including a 49.5% median increase in SIgA (*p* = 0.01), though cortisol remained unchanged. qEEG z-score analysis identified sustained stress reduction, including delta frequency band amplitude increases, high beta decreases, and faster acquisition of sustained alpha states (all *p* < 0.001). Psychological symptoms also improved on all measures. At 6-month follow-up (*N* = 140), PTSD and somatic symptoms significantly improved from baseline, and post-test versus 6-month follow-up results indicated significant increases in happiness and spiritual and physical oneness, along with decreases in depressive symptoms. These findings suggest that autonomic self-regulation and transcendent states may be measured in both biological and psychological dimensions and are associated with pervasive health benefits.

## Introduction

Meditation has been studied extensively for decades and has been found to provide a range of benefits, including stress reduction, mood improvement, and increased health (Baer, 2003). While there are many different schools and forms of meditation such as mindfulness meditation, Zen sitting, mantra repetition, chanting, guided meditation, transcendental meditation, tai chi, clear awareness (Vipassana), lovingkindness (Metta), Hatha yoga, and walking meditation, their commonality is mental training with a goal of wellbeing. This wellbeing takes forms such as stress reduction, improved cognitive function, and the transcendence of ordinary consciousness (Ospina et al., 2007).

Psychological stress downregulates the parasympathetic nervous system which affects several activities directed by the brain, such as respiration, digestion, circulation, and cell metabolism (Kaushik et al., 2020). Stress upregulates the hypothalamus which in turn stimulates the adrenal glands to release cortisol (Seo and Lee, 2010). This further negatively affects the neuro-hormonal levels in the limbic system which over time lead to a cascade of physiological changes in the body such as memory impairments, cognitive deficits, and mood disorders (Yaribeygi et al., 2017). Further, impaired regulation of the amygdala by the pre-frontal cortex is associated with chronic stress (Kaushik et al., 2020). These imbalances also lead to decreased serotonin, which is linked to mood disorders, anxiety, and heightened pain perception (Morena et al., 2016).

Meditation is often associated with improvements in emotional regulation (Robins et al., 2012). This may take the form of enhancement of positive mood (Jain et al., 2007; Tang et al., 2007) and diminished frequency and intensity of dysphoria (Galantino et al., 2005; Chambers et al., 2008; Ding et al., 2014). Meditative practices are associated with improvements in mental health conditions such as anxiety and depression as well as physical symptoms like pain (Shapiro, 2009; Bohlmeijer et al., 2010). Reviews classify guided imagery as a form of mindfulness in that both are forms of selective attention (Chen et al., 2012; Lakhan and Schofield, 2013).

Starting with the earliest investigations of neurofeedback in the 1960s, it has been apparent that an element of meditation involves physiological regulation (Fehmi and Robbins, 2008). Fehmi and Robbins (2008) found that guided imagery could produce the high-amplitude alpha brain waves characteristic of deep meditative states. Emergent disciplines such as functional magnetic resonance imaging (fMRI), electroencephalograph (EEG), salivary endocrine assays (saliva swabs), psychoneuroimmunology, and epigenetics are allowing detailed and systemic maps of the physiological changes associated with meditation to be generated (Church, 2013, 2018).

EEG studies have consistently found that slower alpha frequencies are linked to creativity and relaxation (Klimesch et al., 1998), while beta waves indicate a brain actively involved in cognition and problem solving (Sawant and Jalali, 2010). The characterization of brain waves has advanced to the point where both adaptive and maladaptive patterns can be identified (Kaushik et al., 2020) and a recent study found an association between serum cortisol levels and alpha wave activation (Kamei, 2000). Alpha brainwave activity has further been correlated with decreased pain and discomfort (Palva and Palva, 2011). Individuals practicing transcendental meditation demonstrate increased frontal theta and alpha activity along with decreased levels of anxiety and stress (Chiesa and Serretti, 2010).

After participation in Mindfulness-Based Stress Reduction (MBSR) programs, cortisol is decreased (Matousek et al., 2009) while increased galvanic skin conductance indicates lowered sympathetic nervous system tone (Lush et al., 2009). After the acquisition of mindfulness skills, decreased stress responses are found (Creswell and Lindsay, 2014), as well as quicker recovery to baseline cortisol levels (Brown et al., 2012). After a brief meditation retreat using guided imagery, improvements in both cortisol and SigA were noted (Groesbeck et al., 2018).

As a result of neuroplasticity, the brains of meditators exhibit positive changes over time. The brain regions responsible for emotional regulation, memory, attention, and self-awareness all increase in volume (Fox et al., 2014), while the amygdala – the structure in the midbrain that regulates fear – shrinks (Hölzel et al., 2010). The short-term improvements in stress and mood that result from meditative practices thus eventually become neural pathways as the brain changes it’s signaling priorities and neurogenesis results. In this way, the transient positive states evoked by meditation become traits coordinated by a synchrony of endocrinal, genetic, and neurological activity. However, most studies recruit under 100 participants, lack a control group, measure psychological rather than biological change and lack long-term follow-up; all limitations that indicate gaps in our current understanding of the practice (Creswell and Lindsay, 2014).

The present study was designed to address these shortcomings. It examined changes in psychological and physiological markers among participants in a 4-day guided meditation workshop. The sample size was large, and a 6-month follow up of the experimental group was undertaken. We hypothesized that guided meditation would be associated with improved physiological and psychological functioning, and that these improvements would persist over time. We further hypothesized that these physiological changes could be quantified using biomarkers such as brain waves, cortisol and SIgA, and that we would find an association between improved psychological functioning and changes in physiological indicators.

## Materials and methods

### Participants and procedures

Participants in the study were a convenience sample of 513 individuals out of a total 680 individuals attending a 4-day meditation training. The training, called the Advanced Workshop, was taught by Joseph Dispenza DC. Participants were randomized into two groups (Placebo *N* = 214, Experimental *N* = 299) using an online randomized number generator (randomizer.org); although all 680 attendees at the Advanced Workshop were originally included in the study and randomized into the two groups, 167 attendees failed to provide either or both pre- and post- psychological assessments and were therefore not included in the study analyses. The study design was evaluated for human subject protection by the Ethics Committee of National Institute for Integrative Healthcare (US) and found to present minimal risk to human subjects (Approval #NIIHUS20170109). All participants provided informed consent to participate in the study. Pre-post data analysis was conducted blind to group assignment.

Each day of the Advanced Workshop typically comprised four sessions, each beginning with a lecture on a topic such as the role of belief in the placebo effect, or the role of hormones in stress (Dispenza, 2014) and ending with an extended guided meditation lasting for approximately 1 h. Pre- and post- tests were completed by all participants. The Placebo (PL) group completed the pre-test assessment 1 week before they attended the workshop. After pre-test, they were emailed a link to a website containing poems by 13-century Persian poet and mystic Rumi and instructed to read and contemplate them daily for the period of 1 week. The rationale for this control was that this contemplative practice might be reasonably expected to set up an expectancy effect in the minds of PL group participants. At the end of the week, they completed an online post-test and then attended the workshop. In contrast, the Experimental group completed their pre-test online immediately before the workshop, and their post-test on the final day of the workshop. At 6-month follow-up, psychological assessments were conducted online.

**FIGURE 1:**
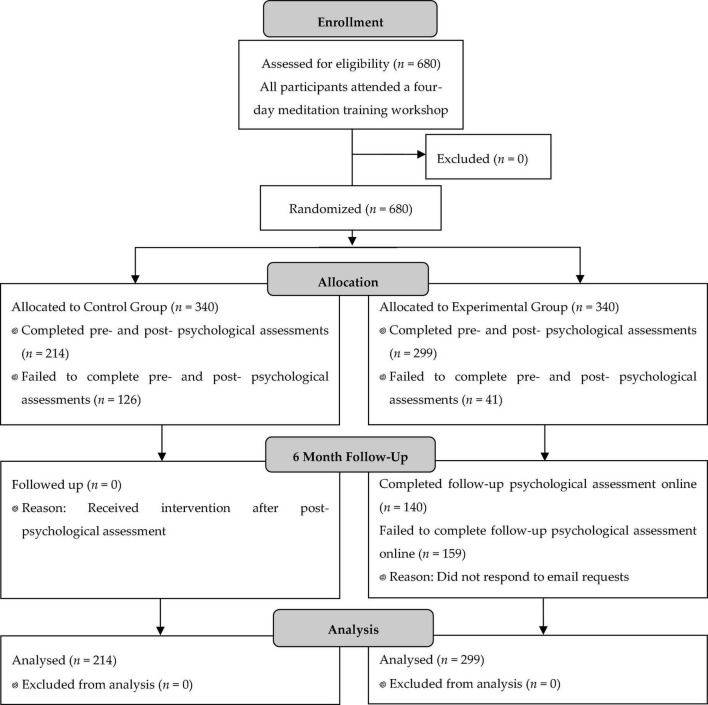
CONSORT flow diagram of study methodology.

A subset of the Experimental group (*N* = 117) was also tested using salivary assays and EEG; this number was limited by the availability of equipment and time available for testing. Salivary immunoglobulin A (SigA) and cortisol were assessed using saliva swabs (Sabre Labs, Capistrano, CA, United States). To eliminate variances due to circadian fluctuations, samples were collected at the same time pre- and post-test (2 p.m.). Following collection, samples were frozen to prevent degradation, and shipped to the lab on dry ice the following day. EEG was measured using the internationally standard 10/20 19 electrode system, and then processed as qEEG data. qEEG can compare brain wave power, relative power, symmetry, coherence between brain regions, phase cross spectrum correlation, burst metrics, and peak frequency. EEG tests were undertaken before the first day of the workshop, and on the afternoon after the final day. Figure 1 outlines a CONSORT Flow Diagram of the methodology.

A repeated research question in EEG studies is whether to use the results from absolute power or relative power. Absolute power refers to the amount of power in a frequency band relative to the total spectrum. Relative power is the percentage of power contained in a frequency band relative to the total spectrum of all frequency bands. Relative power measures which percentage of the overall EEG profile each frequency represents and reflects the proportion of activity in a frequency at a particular location rather than the amplitude of the signal itself as in absolute power. Activity in each frequency band is compared to a normative database to determine the presence of suspected abnormalities. Examining a spectral analysis using relative power, when one frequency band has an increase in its percentage of the total spectrum, the other bands share the remaining areas of the spectrum. This can cause distortion and decrease activity in those frequencies that is not seen in absolute power. This can lead to uncertainty as to whether that band really increased, or only appeared to do so due to the distortion of the other frequencies.

Many studies use relative power because it does not confuse EEG signal with noise, and it is consistent in readings across different software programs. Relative power is insensitive to individual differences that affect EEG. When using absolute power, anatomical differences in individuals such as skull thickness can cause skewed data readings; relative power does not skew data in this way. This has led to relative power being the EEG metric of choice especially for studies involving multiple individuals (Kaiser, 2006).

Relative power records clinically significant change and has the advantage of being able to filter individual results against a normative database. It adjusts for demographics such as gender, dominant hand, and ethnicity. Because absolute power cannot consistently produce the same results across different software packages, and cannot be normed to a database, relative power is used in most research studies. For these reasons, relative power was utilized in the analyses.

### Measures

General psychological and physiological health were assessed using the Patient Health Questionnaire (PHQ) (Kroenke et al., 2002). Questions were drawn from the PHQ9 and PHQ15 forms of the assessment, with the exclusion of the final two questions on the PHQ15 which relate to female obstetrics-gynecology (OB/GYN) function. The Anxiety subscale of the PHQ was analyzed separately. Transcendent states were measured with the Oneness Beliefs Scale (Garfield et al., 2014), created to assess “the inherent unity of all phenomena, or oneness… a central concept of mysticism”. It has two sub-scales, one measuring physical oneness with nature, and the other spiritual oneness, “a short, reliable measure of spirituality not characterized by the language of traditional Western religiousness”. The physical oneness subscale “allows researchers to juxtapose spiritual beliefs with a non-spiritual, materialist counterpart” (Garfield et al., 2014).

While the PHQ9 assesses symptoms and experiences of the previous 2 weeks, pre and posttest were administered about 1 week apart. However, it has been noted that using assessments with time frames longer than the interval between pre and posttest can assess psychological change, because after successful treatment, the way participants perceive the past often changes even though the past itself does not change (Groesbeck et al., 2018). Happiness and pain were assessed using 11-item Likert scales ranging from 0 to 10 with 0 representing minimum and 10 maximum values (Farrar et al., 2001; Abdel-Khalek, 2006). PTSD was assessed with the 2-item form of the PTSD Checklist (PCL) (Lang et al., 2012). All assessments are reliable and valid.

Salivary cortisol is a standard measure of stress (Church et al., 2012). Cortisol rises and falls in a consistent diurnal rhythm, with the typical peak being at 8 a.m. each day in normal individuals, and the trough between midnight and 4 a.m. In those with disrupted endocrine function, such as patients with fibromyalgia, chronic fatigue, or posttraumatic stress disorder, circadian cortisol secretion patterns are dysregulated. In cases of adrenal burnout, cortisol levels may be low throughout the day, and associated with a lack of physical energy and psychological motivation. Successful treatment of psychological conditions such as anxiety and depression can regulate cortisol (Church et al., 2012).

Salivary immunoglobulin A (SIgA) is an important immunological protein present in the body’s mucous membranes. Its primary function is to neutralize pathogens and toxins, a process known as “immune exclusion.” It assists the body in generating mucus in important membranes such as the sinuses and intestinal linings, as well as downregulating inflammation caused by ingested pathogenic bacteria. High levels of SIgA are associated with increased immunity and low levels with decreased immunity. The reduction in stress produced by meditation can result in an increase in beneficial proteins including those responsible for immunity and cell repair. SIgA was therefore measured as a proxy for beneficial cellular processes such as immunity upregulation and the anti-inflammatory response.

Electroencephalograph was measured using a standard 19-electrode array, and then processed as qEEG data. Two measures representing mental quiescence were derived. The first was the time it took for participants to achieve sustained alpha waves (called “time into meditation”). A sustained amplitude of alpha brainwave frequencies, from 9 to 12 Hz, is commonly regarded as a reliable EEG measure of a meditative state. For the purposes of this study, “time into meditation” was defined as the ability of a participant to achieve stable brainwave activity in the alpha frequency band for 15 s or more. On each EEG recording, markers showed the time taken by the participant to reach this sustained alpha threshold. The amplitude and frequency of brain waves waxes and wanes, but when alpha is acquired and consistent for 15 s or longer, this indicates a state change from normal waking consciousness.

The second measure of mental quiescence was the ratio between delta waves and high frequency beta waves (beta 2; 15–30 Hz). An in-depth EEG study examined experienced meditators from five different meditation traditions (Lehmann et al., 2012). The investigators performed a systematic evaluation of the brain activity of participants in all brain regions. From this comprehensive data, they sought common measures that would represent the depth of the meditative state. They found that the two frequency bands in which all five meditation practices produced significant differences were delta and beta 2. Participants in these states reported a loss of a discrete sense of an isolated self and a “subjective experience of non-involvement, detachment and letting go, as well as of all-oneness and dissolution of ego borders.” Beta 2 is associated with stress; highly anxious individuals have large amplitudes of high beta and often exhibit low amplitudes of alpha, theta and delta frequencies. Effective meditation training might therefore be expected result in a reduction in beta 2, increased amplitude of delta, and a concomitant change in the ratio between the two frequencies. Brain wave ratio differences have been observed in other stressed populations (Sheerin et al., 2018; Ulke et al., 2018).

## Results

In order to detect an effect size of Cohen’s *d* = 0.3 with 80% power (alpha = 0.05, two tailed), G*Power indicated a total sample size of 90 participants in a paired sample *t*-test. Therefore the current sample of 513 exceeded this.

Across both PL and Experimental groups, participants at recruitment had a mean age of 54 years and predominantly held a college or graduate degree, with more than two-thirds of total participants being women (PL *N* = 76.17%, Experimental *N* = 76.26%). Despite gender disparity, a chi-squared test of independence (α = 0.05) found no significant differences between groups in relation to gender, χ^2^(1, *N* = 513) = 0.001, *p* = 0.982. There were no statistically significant demographic differences between the PL and Experimental groups.

The summary statistics for pre- and post-test psychological measures are presented in Table 1. As can be observed, the two groups were similar at baseline, with comparisons showing no significant difference on any measure. The within group pre- and post-test comparisons were conducted using paired *t*-tests, displaying significant psychological symptom improvements in the Experimental group from pre-test to post-test in all measures, and improvements in some psychological measures in the PL group (PTSD, PHQ9, PHQ15, and happiness). We further compared the post-test symptom levels between PL and Experimental groups using independent *t*-tests. The Experimental group showed significant psychological improvement compared to the PL group, in PTSD and PHQ15 scores (both *p* < *0.001*), PHQ9 scores (*p* = *0.038*), physical oneness scores (*p* = *0.035*) and pain scores (*p* = *0.001*); additionally, fewer participants demonstrated anxiety symptoms at post-test (*p* < *0.001*). Participants in the Experimental group on average were happier at post-test compared with the PL group (*p* < *0.001*).

**TABLE 1:** Pre- and post-test psychological measures (*N* = 513).

	Placebo			Experimental			Between group comparison	
			
Measure	Pre	Post	*P-value*	Pre	Post	*P-value*	Pre vs pre p-value	Post vs post *p-value*
PTSD mean (SD)	4.59 (1.82)	3.86 (1.66)	<0.001	4.33 (1.85)	3.45 (1.50)	<0.001	0.162	0.019
PHQ9 mean (SD)	4.63 (4.43)	3.84 (3.86)	0.005	3.91 (4.04)	2.71 (3.52)	0.038	0.093	0.007
Anxiety N (%)	121.00 (67.2)	89.00 (65.0)	0.248	150.00 (69.10)	92.00 (52.00)	<0.001	0.766	0.028
PHQ15 mean (SD)	6.47 (4.39)	5.94 (4.34)	0.003	6.11 (4.64)	4.63 (4.09)	<0.001	0.434	0.006
Happiness mean (SD)	7.32 (1.83)	7.62 (1.53)	0.002	7.54 (1.79)	8.22 (1.61)	<0.001	0.230	0.001
Pain mean (SD)	2.50 (2.40)	2.25 (2.32)	0.105	2.36 (2.45)	1.74 (2.32)	0.001	0.560	0.050
Spiritual Oneness mean (SD)	65.28 (13.75)	64.82 (13.30)	0.318	63.96 (16.47)	62.40 (20.30)	0.026	0.393	0.227
Physical Oneness mean (SD)	22.91 (5.76)	23.62 (4.98)	0.307	22.41 (6.42)	22.02 (7.91)	0.035	0.427	0.038

Biological measures were obtained from 117 participants in the Experimental group at pre- and post-test. Due to skews in these measures, non-parametric methods were applied in the form of Wilcoxon signed rank tests to test the pre- and post- difference. The median and interquartile ranges (25–75%) are reported in Table 2 along with the median percentage change for each measure. As can be observed, a significant increase was observed in the SIgA level (49.5%, *p* = 0.01), reduced time to enter meditation (*p* < 0.001), increased delta, and reduced beta 2 (*p* < 0.001 for both). A significant change occurred in the ratio of delta to beta 2 (*p* < 0.001). No significant change was observed in cortisol (16.25%, *p* = 0.888), and no adverse events were reported in either group.

**TABLE 2:** Experimental group pre- and post-test biological measures (*N* = 117).

	Pre			Post			Post-pre% change	
Measure	Median	25%	75%	Median	25%	75%	Median	*P-value*
Cortisol (mcg/dL)	3.00	2.21	4.36	3.13	2.35	4.288	3.04	0.888
SIgA (μg/mL)	47.70	40.15	65.00	52.50	39.89	83.78	5.63	0.01
Time into meditation (s)	99.50	87.00	106.00	79.00	68.00	85.00	−18.42	<0.001
Delta	0.29	−0.47	0.84	2.02	−0.48	4.52	149.31	<0.001
Beta 2	−0.28	−0.92	0.40	−1.45	−3.99	0.89	−123.78	<0.001
Power (Delta/Beta 2)	0.04	−0.92	1.37	−0.68	−1.40	0.63	−62.18	0.033

Follow-up psychological assessments were obtained from the Experimental group at 6 months. Participants maintained their gains on some measures but not others (see Table 3). PTSD remained improved from pre-test to 6-month follow-up (*p* < 0.001), as did PHQ15 scores from pre-test to 6-month follow-up (*p* = 0.002).

**TABLE 3:** Experimental group (*n* = 140) psychological measures at follow-up compared to pre- and post-test.

	Experimental group			*P-value*		
Measure	Pre	Post	Follow-up	Pre vs post	Pre vs follow-up	Post vs follow-up
PTSD mean (SD)	4.33 (1.85)	3.45 (1.50)	3.72 (1.66)	<0.001	<0.001	0.359
PHQ9 mean (SD)	3.91 (4.04)	2.71 (3.52)	3.71 (4.44)	0.038	0.762	<0.001
Anxiety N (%)	150 (69.10)	92 (52.00)	78 (55.7)	<0.001	0.055	0.999
PHQ15 mean (SD)	6.11 (4.64)	4.63 (4.09)	5.40 (4.41)	<0.001	0.002	0.095
Happiness mean (SD)	7.54 (1.79)	8.22 (1.61)	7.75 (1.73)	<0.001	0.174	0.022
Pain mean (SD)	2.36 (2.45)	1.74 (2.32)	2.11 (2.52)	0.001	0.078	0.523
Spiritual Oneness mean (SD)	63.96 (16.47)	62.40 (20.30)	65.49 (13.93)	0.026	0.964	0.015
Physical Oneness mean (SD)	22.41 (6.42)	22.02 (7.91)	22.81 (6.04)	0.035	0.871	0.021

## Discussion

The results of this study demonstrate that guided meditation may lead to both psychological and physiological improvement. Physiological regulation is apparent in the brain function of participants as measured by EEG as well as in immune function as measured by SIgA. Participants were able to acquire and sustain a stable alpha brain state more quickly after the workshop. Their amplitude of the signature wave of stress – beta 2 – diminished. At the same time, their amplitude of the signature wave of a sense of oneness – delta – increased. Their psychological state improved, with PTSD and anxiety symptoms both reducing relative to controls. They self-assessed as happier, with a greater sense of oneness with both nature and the universe. Pain was diminished. These results are consistent with the many other studies of meditation and guided imagery.

The one biological marker that did not improve was cortisol. One possible reason for this is that cortisol synthesis is relatively stable, with a circadian rhythm that does not change markedly over time. Four days may have been insufficient to produce a change in this consistent biorhythm.

The control condition of reading and contemplating Rumi poems was selected because of its anticipated plausibility as a placebo or active control. The improvement of this group on several measures including PTSD, PHQ and happiness suggests that this particular control was in fact an effective intervention. Anecdotal reports provided to the investigators by participants suggested that some had profound transcendental experiences resultant of this activity.

When designing the study, the investigators had difficulty finding an instrument with which to measure transcendent mystical states. After consulting with colleagues with decades of experience in the field, it was found that most assessments measure religious as opposed to spiritual experience. Typical questions relate to frequency of church attendance, prayer, and scripture reading. There is little in the literature to capture the experience of those who are “spiritual but not religious.” Though the Oneness Beliefs Scale was eventually selected, it is our belief that a brief yet robust and representative assessment of transcendent states is required for future research. Until recently, researchers had not asked patients about their spiritual experiences; one of the first studies to do so examined a population of AIDS patients and found that their belief in either a punishing or benevolent universe was the single strongest predictor of the course of the disease (Ironson et al., 2005). Obtaining meaningful data illuminating individual experience is dependent on asking the right questions.

There were a number of limitations to the study. Not all the participants in the Experimental group could receive the biological tests due to limitations of time and equipment. As such, although sufficient to obtain statistical significance, only approximately half of participants completed the biological tests. Additionally, the biological tests were not administered to participants on follow-up because the data was obtained online, thereby rendering the durability of these biological improvements over time unknown. As the PL group was also tested online, no biological measurements of their changes were made, and so no comparison with the Experimental group was possible.

A further limitation is the non-specific effect of any positive social experience such as workshop participation. Demand characteristics and therapeutic allegiance may have played a role in the results. In group settings such as this one, positive emotional contagion can easily spread; at least a portion of the observed effects were likely due to this factor. A further limitation is the limited response rate of participants to the online follow-up. These attrition levels are typical of online post-tests (Church and Brooks, 2010). It therefore cannot be assumed that those who did not respond experienced the same psychological improvements as those who did. Mitigating this limitation, other studies report non-response rates of up to 85% and note that when data are subsequently collected from dropouts by telephone, non-response is not found to bias the outcome (Couper et al., 2007; Ruwaard et al., 2013).

Further research might also illuminate possible dose-dependencies of the duration of meditation experience; would 7 days produce greater change, and do extreme durations like the 3-year retreats of Tibetan Buddhist monks scale in a measurable way? Specialized assessments might be required to determine the effects of very short and very long periods of meditation. We also propose the testing of the beta 2 vs. delta brain wave ratio as a general measure for use in future studies. The field of neurofeedback has been hampered by the lack of such a universally accepted clinical measure.

Despite these limitations, this study adds to the growing body of literature demonstrating the immediate beneficial psychological and physiological effects of meditation. It shows that such experiences may improve immune function, downregulate the brain waves typical of anxiety and stress, upregulate the frequencies associated with relaxation and calmness, improve general measures of physical health, and increase subjective experiences of oneness with the universe and nature. Psychological health is improved, with reduced anxiety, PTSD and pain, and increased happiness. As an easily learned, non-pharmacological intervention without side effects, guided meditation offers both psychological and physiological benefits.

## Data availability statement

The raw data supporting the conclusions of this article will be made available by the authors, without undue reservation.

## Ethics statement

The studies involving human participants were reviewed and approved by the Ethics Committee of National Institute for Integrative Healthcare (US) (Approval #NIIHUS20170109). All participants provided their written informed consent to participate in this study.

## Author contributions

DC: conceptualization, validation, investigation, resources, writing—original draft preparation and review and editing, supervision, and project administration. JF: methodology. AY: formal analysis and visualization. KB: data curation. All authors have read and agreed to the published version of the manuscript.
